# Orbital abscess caused by *Exophiala dermatitidis* following posterior subtenon injection of triamcinolone acetonide: a case report and a review of literature related to *Exophiala* eye infections

**DOI:** 10.1186/s12879-020-05294-y

**Published:** 2020-08-03

**Authors:** Chiharu Iwahashi, Hiroshi Eguchi, Fumika Hotta, Mayu Uezumi, Miki Sawa, Masatomo Kimura, Takashi Yaguchi, Shunji Kusaka

**Affiliations:** 1 Department of Ophthalmology, Faculty of Medicine, Kindai University, 377-2, Ohonohigashi, Osakasayama-shi, Osaka, 589-8511 Japan; 2 Department of Ophthalmology, Sakai City Medical Center, 1-1-1, Ebaraji-cho, Nishi-ku, Sakai City, Osaka, 593-08304 Japan; 3 Department of Diagnostic Pathology, Kindai University Faculty of Medicine, 377-2, Ohnohigashi, Osakasayama, Osaka, 589-8511 Japan; 4 Medical Mycology Research Center Chiba University, 1-8-1, Inohana, Chuo-ku, Chiba-shi, Chiba, 260-8673 Japan

**Keywords:** Orbital abscess, *Exophiala dermatitidis*, Subtenon injection, Voriconazole, *Pseudallescheria boydii*/*Scedosporium apiospermum* complex

## Abstract

**Background:**

Subtenon injection of triamcinolone acetonide (STTA) has been widely adopted in the clinical setting of ophthalmology and its infectious complications are rare. However, orbital abscess following STTA has been reported in seven cases. Furthermore, although eye infections due to *Exophiala* species are uncommon, there have been 19 cases to date. *E. jeanselmei*, *E. phaeomuriformis*, *E. werneckii*, and *E. dermatitidis* have been reported to cause human eye infections; however, to the best of our knowledge, orbital abscess caused by *E. dermatitidis* has not yet been reported. We describe the first documented case of fungal orbital abscess caused by *E. dermatitidis* following STTA. We also review the related literature of orbital abscess following STTA, as well as eye infections caused by the four *Exophiala* species.

**Case presentation:**

The patient was a 69-year-old Japanese woman with diabetic mellitus. She had a macular oedema in her right eye, which occurred secondary to branch retinal vein occlusion. An orbital abscess caused by *E. dermatitidis* occurred 4 months after the second STTA for the macular oedema, which was successfully treated by a surgical debridement and systemic administration of voriconazole.

**Conclusions:**

Our findings in the patient and from our literature survey caution ophthalmologists to the fact that STTA can cause fungal orbital infections, especially in diabetic patients. Furthermore, surgical treatment is one of the most important risk factors.

## Background

*Exophiala dermatitidis*, formerly known as *Wangiella dermatitidis,* is a saprophytic black yeast-like fungus. It is widely distributed in the natural environment, such as soil and dead trees, and it has been isolated from humid indoor habitats such as bathtub water, dishwashers, and humidifiers [1, 2]. Although human infections caused by *E. dermatitidis* are rare, this fungus can occasionally cause subcutaneous and systemic infections. Of the eye infections caused by the *Exophiala* species, we found 19 case reports in the English literature, which identified the species of the causative strains. At present, four species, namely *E. dermatitidis*, *E. jeanselmei*, *E. phaeomuriformis*, and *E. werneckii*, have been reported to be the causative species of eye infections [3–20].

Subtenon injection of triamcinolone acetonide (STTA) has been widely adopted to treat macular oedema secondary to diabetic retinopathy, branch retinal vein occlusion (BRVO), and uveitis. Although infectious complications following STTA are uncommon, endophthalmitis [21], scleritis [22, 23], and orbital abscess [21, 24–28] have been reported. Furthermore, only two cases of fungal orbital abscess following STTA were found to have been reported so far [21–25]. To the best of our knowledge, orbital abscess following STTA caused by *E. dermatitidis* has not been documented.

Herein, we describe the first documented case of orbital abscess caused by *E. dermatitidis* following STTA, which was successfully treated by surgical debridement and systemic voriconazole administration. We have also reviewed related literatures on infectious orbital abscess following STTA, including fungal infections, and of eye infections caused by four *Exophiala* species. A literature search in PubMed was undertaken in March 2020 using the following terms: *Exophiala*, eye infections, keratitis, endophthalmitis, mycetoma, and orbital abscess, and various combinations of these terms.

This study will contribute to alert ophthalmologists to the possibility of fungal infection following STTA and to spread knowledge about eye infections caused by *Exophiala* species.

## Case presentation

A 69-year-old Japanese woman had a macular oedema in her right eye, which occurred secondary to BRVO and was unresponsive to intravitreal injection of anti-vascular endothelial growth factor aflibercept. She was subsequently treated by STTA injection at the referral hospital twice. During the treatments, her eye was anaesthetized with 4% topical lidocaine. A sterilized eyelid speculum was used, and the superior-temporal conjunctiva was incised using sterilized scissors to inject 20 mg of triamcinolone acetonide into the posterior subtenon space. After the injection, the conjunctiva was not sutured because this step is usually unnecessary. These procedures were performed in an outpatient treatment room with wearing sterilized gloves. A 1.5% topical levofloxacin ophthalmic solution was administered four times daily for 3 days post-injection. Four months after the second injection, the patient noted a conjunctival mass that became progressively larger with a little pain and redness. She was then referred to Kindai University with a clinical diagnosis of orbital fat herniation.

**Fig. 1:**
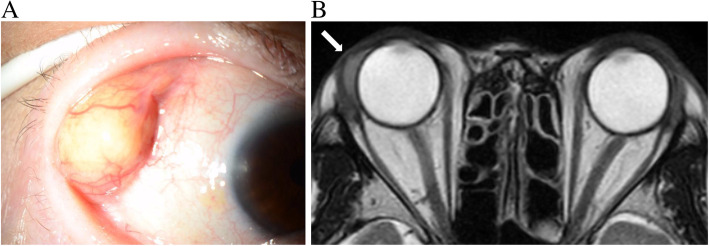
**a**. An image of anterior segments. Well-demarcated mass mimicking herniated orbital fat was observed in the superior-temporal quadrant. **b**. Magnetic resonance imaging of the orbit. A hypo-intense lesion on T2-weighted images at the temporal peribulbar area was found

During her first visit, best-corrected visual acuity (BCVA) was 6/30 in the right eye and 6/6 in the left eye. Intraocular pressure was normal in both eyes. Slit-lamp microscopic examination revealed no inflammation in both the anterior chamber and vitreous. A soft, well-demarcated mass mimicking herniated orbital fat was observed in the super-temporal quadrant, which corresponded to the posterior STTA site (Fig. 1a). Magnetic resonance imaging (MRI) of the orbits revealed a hypo-intensity lesion on T2-weighted imaging at the super-temporal peribulbar area (Fig. 1b). Her white cell count and C reactive protein levels were normal.

**Fig. 2:**
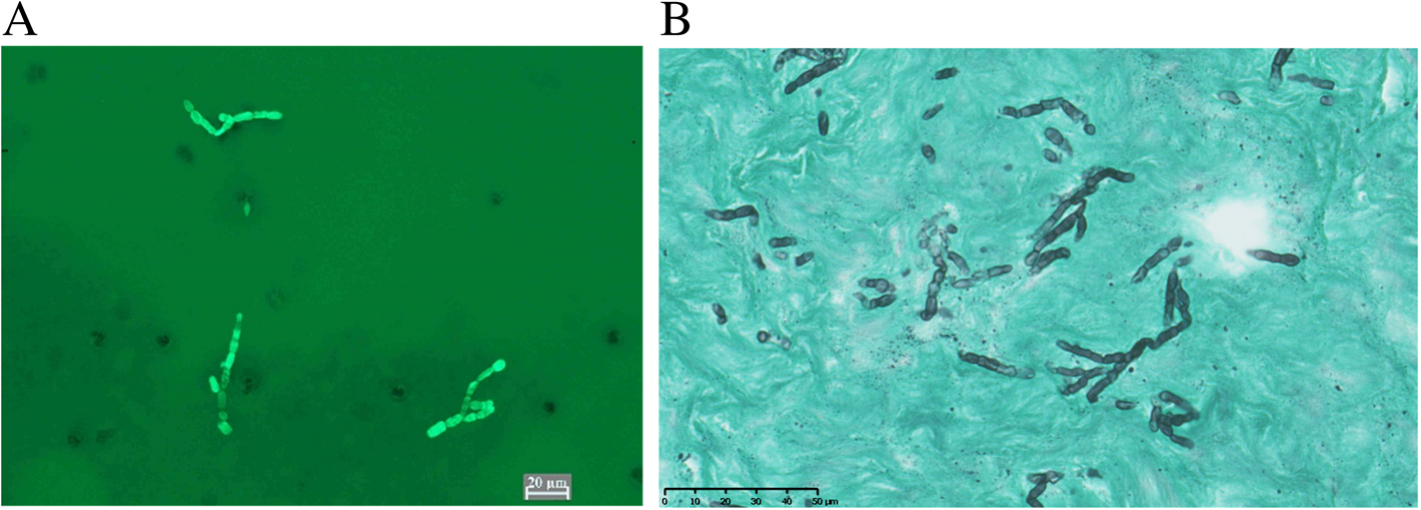
**a**. Fungiflora Y staining of the pus. Fungi were found. **b**. Pathological examination of the abscess by Grocott’s staining. Fungi were still found in the excised abscess

To excise the conjunctival mass, first, we made an incision in the conjunctiva, and found that yellowish pus was discharged. The local administration of antibiotics was performed empirically based on the presumed diagnosis of bacterial infection. Although a strain of dematiaceous fungi was isolated from the pus 1 month later, the antifungal drug susceptibility of the strain was not determined at that time. We did not immediately administer antifungal drugs because there were no significant changes in clinical findings initially. The abscess was debrided again 2 months after the surgery through a superior forniceal incision because the orbital abscess at the same lesion had developed gradually. During the second surgery, the wound was irrigated with 0.1% amphotericin B after three large abscesses were removed. Postoperatively, she was treated with 400 mg/day of oral voriconazole because of Fungiflora Y staining of the pus (Fig. 2a) and the presence of a large amount of fungi by pathological examination of the abscess by Grocott’s staining (Fig. 2b).

**Fig. 3:**
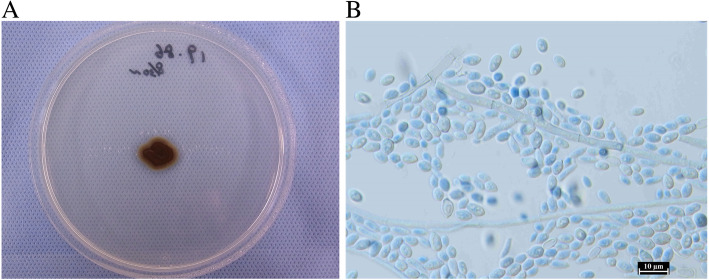
**a**. An image of a large colony on potato dextrose agar medium. The pigmented, moist form of a yeast-like colony 14 days after inoculation is shown

We obtained the pathogenic strain from the abscess during the second surgery and observed it macroscopically and microscopically. The colony on potato dextrose agar medium (PDA; Difco, Becton, Dickinson and Company, Sparks, USA) had a diameter of 10 mm 14 days after inoculation and with incubation at 25 °C. The colony appeared as a moist-form pigmented yeast-like colony (Fig. 3a). The slide culture using lactophenol cotton blue staining revealed cylindrical conidogenous cells producing ellipsoidal conidia (Fig. 3b). From the Basic Local Alignment Search Tool search using the sequence of the ribosomal RNA gene internal transcribed spacer domain base, the sequence (the accession number LC566592 in the DDBJ) of this strain showed 100% similarity to those of some strains of *E. dermatitidis*. The isolate was thus identified as *E. dermatitidis* on the basis of morphology and phylogeny. The pathogenic strain in the current case was deposited at the Medical Mycology Center, Chiba University in Japan, as IFM 65961.

Minimum inhibitory concentrations (MICs) of several antifungal drugs and the minimal effective concentration of micafungin against the strain were determined using of the broth dilution method according to M38-A2 of the Clinical and Laboratory Standard Institute (Table 1). The inhibitory concentrations of amphotericin B, itraconazole, voriconazole, and miconazole against the pathogenic strain were found to be low. Based on these results, topical and systemic voriconazole were administered for 3 months. The patient’s serum beta-D-glucan levels were normal during the follow-up periods since the first surgery, and no recurrence was observed after the second drainage.

**Table 1:** Susceptibility of antifungal agents for *Exophiala dermatitidis*

Antifungal agents	MIC/MEC (μg/mL)
Micafungin	4
Caspofungin	16
Amphotericin B	1
Flucytosine	4
Fluconazole	16
Itraconazole	1
Voriconazole	0.5
Miconazole	1

*MEC* minimum effective concentration, *MIC* minimum inhibitory concentration

## Discussion and conclusion

Periocular infection is a rare complication following STTA with a reported incidence of 0.04% in the Japanese population [29]. As listed in Table 2, two of the six reported infectious orbital abscesses following STTA were caused by fungi, whereas the rest were caused by bacteria. The causative fungi have been identified as the *Pseudallescheria boydii*/*Scedosporium apiospermum* complex. At present, the reason why only *Scedosporium* species have been reported as the causative fungi of orbital abscesses following STTA is uncertain. However, *S. apiospermum* is classically known to cause trauma-associated infections in healthy individuals, and it certainly can cause infections in immunocompromised hosts [30]. Its abundance has also been correlated with human impact on environments [31], particularly with increasing level of diesel fuels and elevated temperatures [32]. Given that urban soils can reach high temperatures even in countries with temperate climates [32], the ocular surface of individuals living in urban areas with temperate climates is expected to be more exposed to *S. apiospermum*.

**Table 2:** Reported cases of orbital abscess after posterior subtenon injection of triamcinolone acetonide (STTA)

Age/ Sex	Year reported	Comorbidities		Duration^a^	Causative pathogen	Reference
		Focal	Systemic			
90/F	2004	BRVO, ME	None	3 weeks	*Staphylococcus aureus*	[24]
62/F	2007	DME	DM	2 months	*Pseudallescheria boydii*	[25]
50/M	2007	Uveoscleritis	Behçet disease, DM	2 weeks	*Nocardia* species	[26]
54/F	2008	DME	DM, HT	3 days	Gram-positive cocci	[27]
58/M	2009	DME	DM	3 months	*Scedosporium apiospermum*	[21]
57/M	2017	CME	Atopic dermatitis	6 weeks	*Staphylococcus aureus*	[28]
69/F	2020	BRVO, ME	DM	4 months	*Exophiala dermartitidis*	The current case

*F* female, *M* male, *BRVO* branch retinal vein occlusion, *DME* diabetic macular oedema, *CME* cystoid macular oedema, *DM* diabetes mellitus, *HT* hypertension

^a^Duration denotes periods from the last STTA

Interestingly, as listed in Table 3, most of the reported cases of eye infection caused by *Exophiala* had a history of certain surgical interventions [3, 4, 6–8], which may have caused trauma to human tissues. *E. dermatitidis* can be isolated from environments where human impact is present, similarly to *S. apiospermum* [33]. Both reported patients with fungal abscess following STTA, along with the present study subject, had diabetes mellitus, which might have made them susceptible to fungal eye infection because of local immunosuppressive conditions. Therefore, diabetes mellitus should be considered as the highest risk factor of fungal orbital infection following STTA, and *E. dermatitidis* and *S. apiospermum* should be considered as the causative pathogens.

**Table 3:** Reported cases of eye infection caused by *Exophiala* as classified by species

Species	Diseases	Age/Sex	Outcome	Possible etiologies	Year	Country	Reference
*E. dermatitidis*	Orbital abscess	69/F	Healed	STTA, DM	2020	Japan	The current case
	Endophthalmitis	59/M	Healed	Cataract surgery	2018	India	[3]
	Endophthalmitis	60/M	Not healed	PK	2014	USA	[4]
	Subconjunctival mycetoma	44/F	Healed	unknown	2010	Hong Kong	[5]
	Keratitis	52/F	Healed	LASIK	2006	USA	[6]
	Keratitis	75/M	Not healed	PK, steroid	2006	Taiwan	[7]
	Keratitis	31/M	Not healed	PK	1999	France	[8]
	Keratitis (corneal abscess)	35/M	Healed	Recklinghausen’s disease	1990	Czechoslovakia	[9]
*E. jeanselmei*	Keratitis	41/M	Healed	Plant injury	2013	USA	[10]
	Subconjunctival mycetoma	76/M	Not healed	STTA	2009	USA	[11]
	Keratitis	39/F	Healed	LASIK, rock-climbing	2008	USA	[12]
	Keratitis	58/F	Healed	Trauma	2002	Israel	[13]
	Endophthalmitis	67/M	Not healed	Cataract surgery, steroid	1999	Brazil	[14]
	Endophthalmitis	52/F	Not healed	Cataract surgery, DM	1999	Brazil	[14]
	Keratitis	42/M	Not healed	–	1993	Saudi Arabia	[15]
	Endophthalmitis	–	Not healed	Trauma	1983	–	[16]
*E. phaeomuriformis*	Keratitis	67/M	Healed	KPro, CL	2018	USA	[17]
	Keratitis	81/F	Healed	Laser treatment, CL	2018	Sweden	[18]
	Keratitis	84/F	Healed	PK	2017	USA	[19]
*E. werneckii*	Endophthalmitis	83/F	Healed	Not determined	2000	USA	[20]

*F* female, *M* male, *STTA* subtenon injection of triamcinolone acetonide, *PK* penetrating keratoplasty, *LASIK* laser in situ keratomleusis, *DM* diabetes mellitus, *KPro* keraoprosthesis, *CL* contact

The possibility of serum fungal infection in the present case was very low because her serum beta-D-glucan level was within the normal range throughout the follow-up periods since the first surgery. We speculate that the cause of exogenous origin was direct contact with polluted soil or rubbing the eyes with contaminated fingers after handling polluted indoor habitats, thereby allowing the pathogenic strain to access the subtenon space via conjunctival incision early after the STTA procedure. Given the extremely low frequency of infectious complications following STTA performed in the referral hospital and with the study being the only case out of more than 2000 STTA cases similarly conducted for more than 20 years, we speculate the iatrogenic transmission route to be very unlikely. We presume that this patient had a certain affinity to *E. dermatitidis*, probably from the polluted humid indoor environments in her house; however, no interview about her living environment was done. Alternatively, a strain of *E. dermatitidis* could have colonized on her ocular surface, given the fact that yeasts, including *E. dermatitidis*, have been cultured from the corneas of healthy cadavers, but more yeasts were isolated from the corneas of diabetic cadaver [34]. It should be noted that STTA, through small conjunctival incisions, possibly causes fungal orbital infection, even if sterilized instruments were used following ocular surface disinfection.

To date, four *Exophiala* species, namely *E. jeanselmei*, *E. phaeomuriformis*, *E. werneckii*, and *E. dermatitidis* have been isolated from human eye infections. We have listed all the studies published in English that performed species-level identification of the causative strain (Table 3). There is no tendency for certain species of *Exophiala* to have a higher affinity towards certain parts of the eye. However, *E. jeanselmei* and *E. dermatitidis* have higher affinities to the human eye than the other species do [3–16]. Interestingly, in the past 5 years, *E. phaeomuriformis* has been reported as a causative strain of eye infections. In 2018, the first case of *E. phaeomuriformis* keratitis was reported from subarctic region [18]. The emergence of *Exophiala* spp., which are pathogenic to the human eye, suggests that the increase in *Exophiala* eye infections might be related to global warming given that *E. phaeomuriformis* and *E. dermatitidis* grow at 45–47 °C [35]. To determine whether this trend would continue, case accumulation is required. Regarding the ocular involvement of *E. dermatitidis*, keratitis, subconjunctuval mycetoma, and endophthalmitis have been reported. Four of six *Exophiala* endophthalmitis cases did not heal, which led to poor visual outcome. If not treated properly, endophthalmitis may occur secondary to orbital abscess; therefore, the surgical debridement of a fungal abscess and appropriate administrations of antifungal agents were effective in the current case. As shown in Table 2, bacterial orbital abscess develops within 4–6 weeks, whereas fungal orbital abscess develops after 2–3 months. In the present case, orbital abscess occurred 3 months after the second STTA and recurred 2 months after the first surgery. Hence, careful monthly monitoring is required after STTA. Diagnostic imaging using MRI should be performed if orbital abscess is suspected. If necessary, a prompt surgical approach should be considered because *E. dermatitidis* can cause infection in the central nervous system, leading to fatal consequences [36, 37].

The study of in vitro antifungal susceptibilities of environmental *Exophiala* isolates has shown that the most active antifungal agents are voriconazole and terbinafine, followed by posaconazole, itraconazole and amphotericin B, and that fluconazole has no meaningful antifungal activity against *Exophiala* [38]. In the current case, the recurrence of orbital abscess was observed without antifungal systemic treatment in the postoperative period of the first surgery. Therefore, after the second surgical debridement, we had administered systemic voriconazole for 3 months based on the susceptibility of antifungal drugs and no recurrence was observed. Prolonged systemic antifungal administration is required for the treatment of fungal orbital infection.

In conclusion, ophthalmologists should be aware that STTA can cause fungal orbital infections, especially in diabetic patients. Surgical treatment is one of the highest risk factors. *E. dermatitidis*, along with the *Pseudallescheria boydii*/*Scedosporium.*

*apiospermum* complex, should be considered as a causative pathogen. The monthly monitoring of clinical findings obtained using diagnostic imaging and a surgical debridement in an optimal period is crucial. Systemic administration of voriconazole after the surgery is also recommended.

## Data Availability

The datasets for the current study are available from the corresponding author on reasonable request.

## Abbreviations

STTA: Subtenon injection of triamcinolone acetonide
BCVA: Best-corrected visual acuity
MRI: Magnetic resonance imaging
MIC: Minimum inhibitory concentration
